# Association of serum sodium and risk of all-cause mortality in patients with chronic kidney disease: A meta-analysis and sysematic review

**DOI:** 10.1038/s41598-017-16242-3

**Published:** 2017-11-21

**Authors:** Liguang Sun, Yue Hou, Qingfei Xiao, Yujun Du

**Affiliations:** 1 Institute of Immunology, The First Hospital of Jilin University, 130021 Changchun, China; 2 Department of Nephrology, The First Hospital of Jilin University, 130021 Changchun, China

## Abstract

Studies on the association of dysnatraemia with all-cause mortality risk in chronic kidney disease (CKD) patients have yielded inconsistent results. This meta-analysis aimed to evaluate the association of hyponatremia or hypernatremia with all-cause mortality risk in CKD patients. An electronic literature search was performed in Web of Science, Pubmed and Embase databases from inception to March 2017 for available observational studies evaluating the association of dysnatraemia with all-cause mortality risk in CKD patients. Pooled hazard risk (HR) with 95% confidence interval (CI) was calculated for hyponatremia or hypernatremia *vs*. normonatremia. Seven studies that enrolled 742,979 CKD patients were identified. Baseline hyponatremia (HR 1.34; 95% CI: 1.15–1.57), and not hypernatremia (HR 1.12; 95%: CI 0.93–1.34), was independently associated with increased risk of all-cause mortality, when compared than the normonatremia category. In time-dependent analyses, both time-averaged hyponatremia (HR 1.65; 95% CI: 1.27–2.15) and hypernatremia (HR 1.41; 95% CI: 1.20–1.65) had a higher independent risk of all-cause mortality. Furthermore, subgroup analyses by type of patients, study design, sample size and follow-up duration revealed similar results across most of these analyses. Baseline hyponatremia and time-dependent hyponatremia or hypernatremia were independently associated with increased all-cause mortality risk in CKD patients.

## Introduction

Chronic kidney disease (CKD) is an increasing global public health concern^1^. End-stage renal disease (ESRD) is the chronic and progressive decline in kidney function. More than two million people suffer from ESRD worldwide^2^. Furthermore, the number of patients with ESRD receiving maintenance hemodialysis or peritoneal dialysis continues to increase worldwide^3^. Given that the mortality rate of CKD patients with or without dialysis is unacceptably high^4,5^, early risk stratification for mortality is crucial in these populations.

Dysnatremia is the most common electrolyte abnormality in clinical practice. Clinically, normal serum sodium level in humans ranges within 135–144 mmol/L^6^. Patients with CKD tend to develop dysnatremia mainly due to their diminished ability to maintain water homeostasis^7^. Hyponatremia and hypernatremia are relatively frequent electrolyte abnormalities in patients with advancing stages of CKD, who are undergoing dialysis^8^. Several^9–17^ but not all^18,19^ epidemiologic studies reported that hyponatremia is associated with increased all-cause mortality in no-dialysis CKD and maintenance dialysis patients. Similarly, the association of hypernatremia with all-cause mortality risk has also yielded inconsistent results^9,11–14,19^.

To the best of our knowledge, no meta-analysis has addressed the association of baseline and time-dependent dysnatremia with subsequent all-cause mortality risk among CKD patients. Given the conflicting findings in these available studies, we conducted a meta-analysis to investigate whether dysnatremia (hyponatremia and hypernatremia) was an independent predictor of all-cause mortality in CKD patients with or without dialysis.

## Results

### Literature search and study characteristics

**Figure 1:**
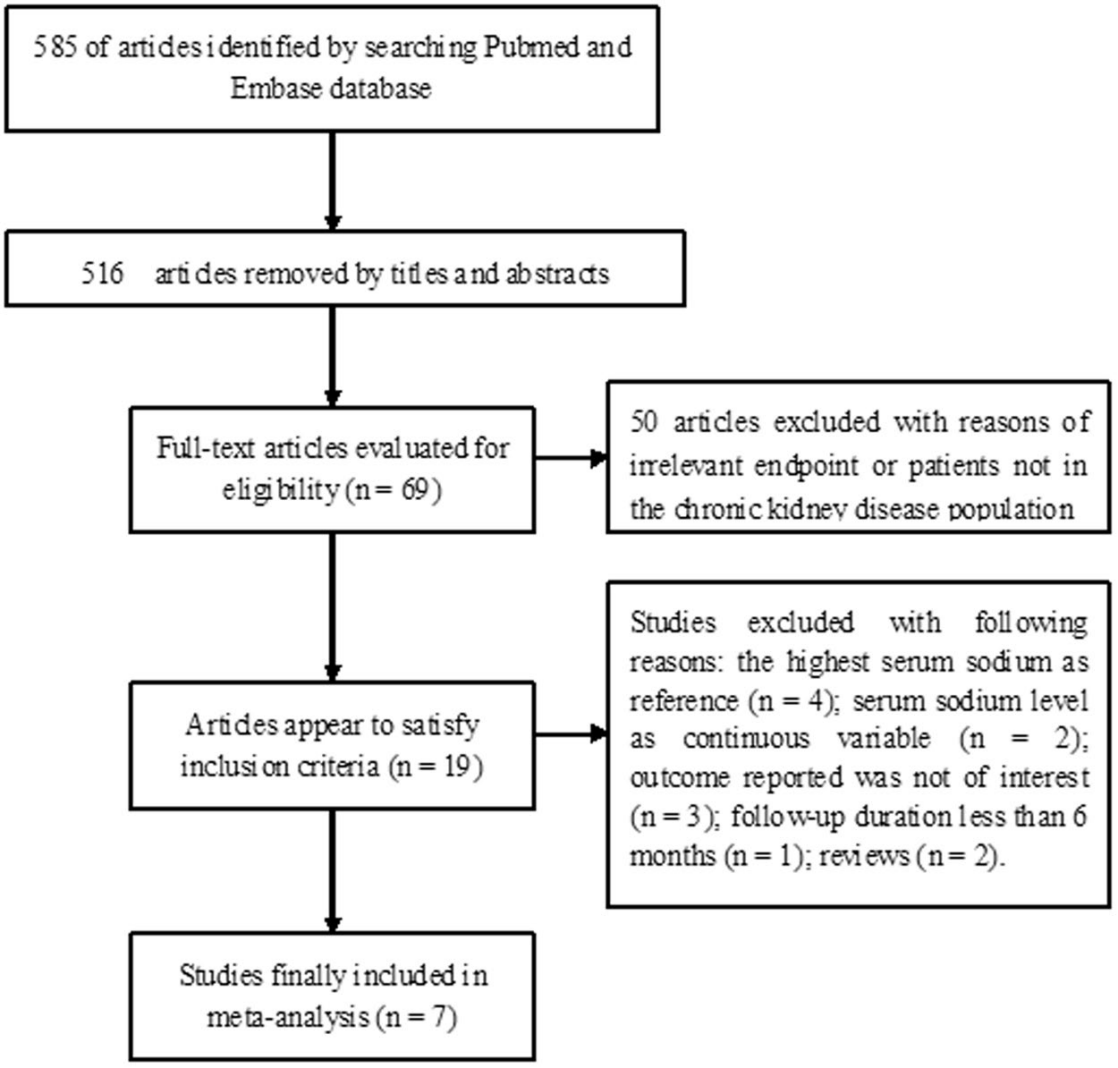
Flow chart of the study selection process.

The initial literature search yielded 585 articles. Among these retrieved articles, 578 articles were excluded for different reasons (Fig. 1). Thus, seven studies^9–14,19^ that comprised of 742,979 CKD patients were included in the meta-analysis. Table 1 presents the baseline characteristics of the included studies. Among the seven articles, four studies^9,11,14,19^ were limited to non-dialysis CKD patients, two studies^10,12^ enrolled hemodialysis patients, and one study^13^ included peritoneal dialysis patients. Furthermore, five studies^9,10,12–14^ were conducted in the United States, Korea^19^ and the United Kingdom^11^, in which each country contributed one study. All seven studies followed an observational design, in which four studies were retrospective cohorts^9,12–14^ and three studies were prospective cohorts^11,19^. Furthermore, four studies^9–11,14,19^ defined hyponatremia as serum sodium <136 mmol/L and three studies^10,12,13^ defined hyponatremia as serum sodium <135 mmol/L. The cut-off definition of hypernatremia was ≥144 mmol/L and ≥145 mmol/L in these included studies. All included studies were deemed as high quality, with a rank of 6-8 stars in the Newcastle–Ottawa Scale (NOS) (Table 2).

**Table 1:** Summary of clinical studies included in meta-analysis.

Study/year	Country	Design	Type of patients	Baseline eGFR	Sample size (% male)	Age/range Mean ± SD	Dysnatraemia definition/number	Number of death/HR (95% CI)	Follow-up duration	Adjustment for covariates
Kovesdy *et al*. 2012^9^	USA	Retrospective cohort study	Non-dialysis CKD	55.2 ± 19.3 ml/min/1.73 m^2^	655, 493 (97.2)	73.9 ± 9.8	Na < 136 mmol/L (85,855); Na > 145 mmol/L (350)	Total death: 193,956 Baseline-Na 1.06 (1.03–1.10); L 1.02 (0.93–1.11); H TA-Na 1.32 (1.15–1.51); L 1.20 (1.10–1.31); H	5.5 years	Age, gender, race, geographic location, DM, CVD, CHF, liver disease, malignancy, depression, CCI, SBP, eGFR, serum albumin, AKP, AST, ALT, total bilirubin, hemoglobin, glucose, and WBC
Nigwekar *et al*.2013^10^	USA	Prospective cohort study	HD	10.5 ± 6.2ml/min/1.73 m^2^	6,053 (54.4)	62.5 ± 15.2	Na < 135 mmol/L (775)	Total death: 965 Baseline-Na 1.42(1.19–1.69)	12 months	Age, race, sex, DM, hypertension, CAD, catheter access, facility mortality statistic, BMI, serum albumin, bicarbonate level.
Han *et al*. 2015^19^	Korea	Prospective cohort study	Non-dialysis CKD	25.5 ± 10.7 ml/min/1.73 m^2^	2,141 (55.2)	63.5 ± 14.9	Na ≤ 135 mmol/L (135); Na ≥ 144 mmol/L (350)	Total death: 182	1.8 years	Age, gender, race, eGFR, SBP, hemoglobin, CVD, serum albumin
								Baseline-Na		
								1.74 (0.84–3.60); L		
								2.01(1.21–3.34); H		
								TA-Na		
								0.93 (0.44–1.97); L		
								1.53 (0.92–2.55); H		
Chiu *et al*. 2016^11^	UK	Prospective cohort study	Non-dialysis CKD	32.8 ± 15.9 ml/min/1.73 m^2^	2,093 (62.6)	56–75	Na ≤ 135 mmol/L (142);	Total death: 684	41 months	Age, gender, smoking, DM, previous MI, heart failure, SBP, eGFR, serum albumin, use of renin-angiotensin blocker or diuretics
							Na ≥ 145 mmol/L (134)	Baseline-Na		
								1.35 (1.02–1.78); L		
								1.15 (0.84–1.57); H		
								TA-Na		
								2.15 (1.59–2.91); L		
								1.47 (0.93–2.38); H		
Rhee *et al*. 2016^12^	USA	Retrospective study	HD	—	27,180 (57)	63 ± 15	Na < 136 mmol/L (2,501);	Total death: 7,562	1.4 years	Age, sex, race/ethnicity, primary insurance, vascular access, comorbidities, IDWG, Kt/V, residual urea clearance, BMI, serum albumin, creatinine, total iron binding capacity, ferritin, iron saturation, bicarbonate, PTH, calcium, phosphorus, hemoglobin, glucose, WBC, BUN, and normalized protein catabolic rate.
							Na ≥ 144 mmol/L (549)	Baseline-Na		
								1.39 (1.21–1.59); L		
								0.84 (0.71–0.99); H		
								TA-Na		
								1.64(1.34–2.02); L		
								1.47 (1.26–1.71); H		
Ravel *et al*. 2016^13^	USA	Retrospective study	PD	—	4,687 (55)	58 ± 15	Na < 136 mmol/L (399);	Total death: 649	11.9 months	Age, sex, race/ethnicity, primary insurance, baseline comorbidities, serum albumin, creatinine, total iron binding capacity, calcium, phosphorus, PTH, ferritin, iron saturation, hemoglobin, WBC, peritoneal Kt/v, renal Kt/V, use of automated PD during the baseline quarter or anytime
							Na ≥ 144 mmol/L (170)	Baseline-Na		
								1.48 (1.14–1.92); L		
								1.03 (0.65–1.65); H		
								TA-Na		
								1.52(1.22–1.89); L		
								1.17 (0.73–1.88); H		
Huang *et al*. 2017^14^	USA	Retrospective study	Non-dialysis CKD	48.0 ± 10.2 ml/min/1.73 m^2^	45,333 (44.6)	71.9 ± 11.9	Na < 136 mmol/L (3,626);	Total death: 11,715	3.6 years	Age, gender, smoking, BMI group, eGFR, DM, hypertension, cerebrovascular disease, CAD, CHF, hyperlipidemia, malignancy, ACEI/ARB, beta blocker, diuretics, albumin, hemoglobin, serum bicarbonate and liver disease
							Na > 145 mmol/L (532)	Baseline-Na		
								1.39 (1.32–1.48); L		
								1.31 (1.14–1.51); H		
								TA-Na		
								2.24 (2.14–2.35); L		
								1.66 (1.44–1.91); H		

Abbreviations: HD, hemodialysis; PD, peritoneal dialysis; L, low; H, high; HR, hazard ratio; CI, confidence interval; CVD, cardiovascular disease; CAD, coronary artery disease; CHF, congestive heart failure; DM, diabetes mellitus; BMI, body mass index; CKD, chronic kidney disease; MI, myocardial infarction; eGFR, estimated glomerular filtration rate; WBC, white blood cell count; CCI, Charlson Comorbidity Index; BUN, blood urea nitrogen; PTH, parathyroid hormone; TC, total cholesterol; TG, triglyceride; SBP, systolic blood pressure; AST, aspartate aminotransferase; ALT, alanine aminotransferase; AKP, alkaline phosphatase; TA-Na,time-averaged serum sodium; ACEI, angiotensin-converting enzyme inhibitor; ARB, angiotensin receptor blocker.

**Table 2:** quality assessment of studies included in meta-analysis

Study/Year	Representativeness of the exposed cohort	Selection of the non exposed cohort	Ascertainment of exposure	Demonstration that outcome was not present at study start	Comparability of cohorts on the basis of the design or analysis	Assessment of outcome	Enough follow-up periods (≥3 years)	Adequacy of follow-up of cohorts	Overall NOS
Kovesdy *et al*. 2012 ^9^	★	★	★	★	★	★	★	★	**8**
Nigwekar *et al*. 2013^10^	★	★	★	★	★	★			**6**
Han *et al*. 2015^19^	★	★	★	★	★	★			**6**
Chiu *et al*. 2016^11^	★	★	★	★	★	★	★		**7**
Rhee *et al*. 2016^12^	★	★	★	★	★	★		★	**7**
Ravel *et al*. 2016^13^	★	★	★	★	★		★	★	**7**
Huang *et al*. 2017^14^	★	★	★	★	★	★	★	★	**8**

### The association of hyponatremia with all-cause mortality

**Figure 2:**
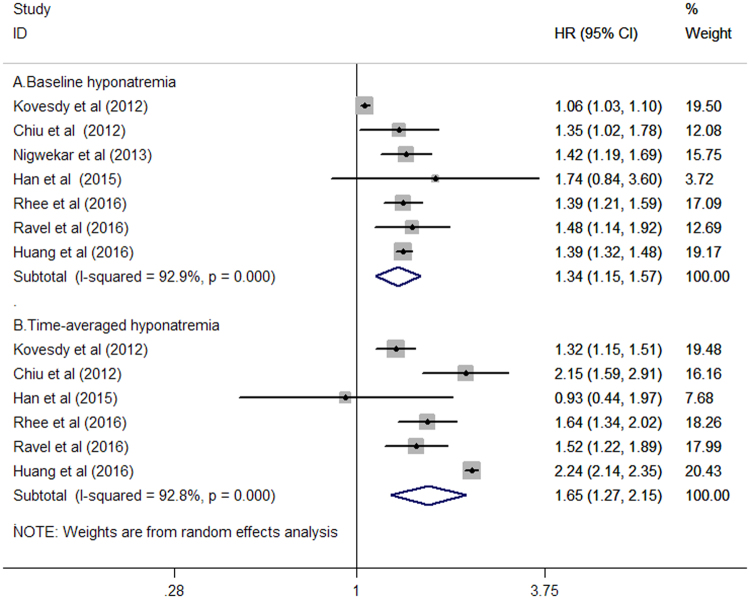
Forest plots showing HR and 95% CI of all-cause mortality comparing baseline hyponatraemia (**A**) or time-averaged hyponatraemia (**B**) to normonatremia in a random effect model.

All the included studies^9–14,19^ evaluated the association of baseline hyponatremia and risk of all-cause mortality. In the time-dependent analyses of hyponatremia, six studies^9,11–14,19^ assessed this association. As shown in Fig. 2A, there was significant heterogeneity across these included studies (*I* ^2^ = 92.9%, *P* < 0.001). The meta-analysis revealed that patients with baseline hyponatremia were associated with a higher risk of all-cause mortality (hazard ratio [HR]: 1.34; 95% confidence interval [CI]: 1.15–1.57) compared with patients with normonatremia in a random effects model. Furthermore, there was no evidence of publication bias according to Begg’s test (*P* = 0.368) and Egger’s test (*P* = 0.151). The sensitivity analysis, in which an individual study was remove at a time, revealed that pooled HR varied within 1.32–1.40 and low 95% CI varied within 1.11–1.33. Similarly, patients with time-averaged hyponatremia was associated with higher risk of all-cause mortality (HR: 1.65; 95% CI: 1.27–2.15; Fig. 2B) compared with patients with normonatremia in a random effects model, and there was significant heterogeneity across studies (*I* ^2^ = 92.8%; *P* < 0.001). Begg’s test (*P* = 1.000) and Egger’s test (*P* = 0.117) did not reveal any evidence of publication bias. The sensitivity analysis indicated that pooled HR varied within 1.54–1.78 and low 95% CI varied within 1.16–1.41.

### The association of hypernatremia and all-cause mortality

**Figure 3:**
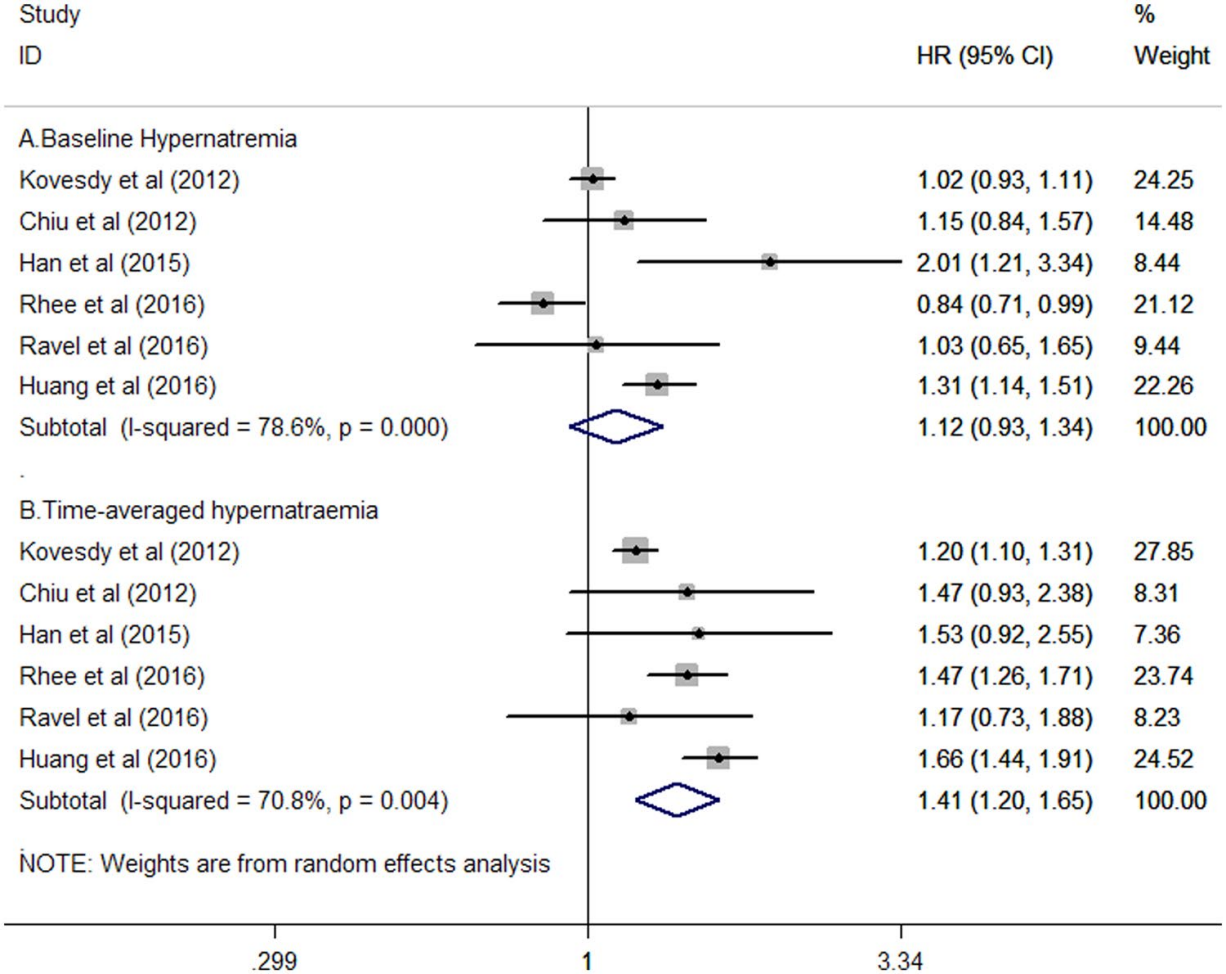
Forest plots showing HR and 95% CI of all-cause mortality comparing baseline hypernatraemia (**A**) or time-averaged hypernatraemia (**B**) to normonatremia in a random effect model.

Six studies^9,11–14,19^ reported the association of baseline and time-averaged hypernatremia with all-cause mortality risk. The meta-analysis revealed that patients with baseline hypernatremia were not significantly associated with increased risk of all-cause mortality (HR: 1.12; 95% CI: 0.93–1.34; Fig. 3A) compared with patients with normonatremia in a random effects model. Furthermore, there was significant heterogeneity across these included studies (*I* ^2^ = 78.6%; *P* < 0.001). Moreover, Begg’s test (*P* = 0.707) and Egger’s test (*P* = 0.523) suggested no evidence of publication bias. The sensitivity analysis revealed that pooled HR varied within 1.06–1.17 and low 95% CI varied within 0.88–0.99. In the time-dependent analysis of hypernatremia, patients with time-averaged hypernatremia was associated with higher risk of all-cause mortality (HR: 1.41; 95% CI: 1.20–1.65; Fig. 3B) compared with patients with normonatremia in a random effects model, and there was substantial heterogeneity across these studies (*I* ^2^ = 70.8%; *P* = 0.004). Furthermore, there was no evidence of publication bias based on the results of Begg’s test (*P* = 1.000) and Egger’s test (*P* = 0.522). The sensitivity analysis indicated that pooled HR varied within 1.32–1.54 and low 95% CI varied within 1.13–1.40.

### Subgroup analyses

The stratified analysis revealed similar results across most of the subgroups, indicating a consistent association between dysnatremia and all-cause mortality (Tables 3 and 4).

**Table 3:** Subgroup analysis of hyponatraemia and all-cause mortality.

Subgroup	No. of studies	Pooled HR	95%CI	Heterogeneity between studies
**1. Baseline hyponatraemia**				
Sample sizes				
≥10,000	3	1.26	1.02–1.57	p < 0.001; I^2^ = 97.3%;
<10,000	4	1.43	1.26–1.62	p = 0.915; I^2^ = 0.0%
Study design				
Prospective	3	1.41	1.22–1.63	p = 0.810; I^2^ = 0.0%;
Retrospective	4	1.3	1.07–1.58	p < 0.001; I^2^ = 96.1%
Follow-up duration				
≥2 years	3	1.24	0.99–1.56	p < 0.001; I^2^ = 97.0%
<2 years	4	1.42	1.28–1.56	p = 0.921; I^2^ = 0.0%
Type of patients				
Dialysis	3	1.41	1.28–1.56	p = 0.914; I^2^ = 0.0%
Non-dialysis CKD	4	1.27	1.02–1.59	p < 0.001; I^2^ = 95.6%
**2. Time-averaged hyponatraemia**				
Sample sizes				
≥10,000	3	1.7	1.17–2.48	p < 0.001; I^2^ = 96.5%;
<10,000	3	1.61	1.14–2.29	p = 0.057; I^2^ = 65.1%
Study design				
Prospective	2	1.52	0.68–3.42	p = 0.042; I^2^ = 75.8%;
Retrospective	4	1.66	1.21–2.26	p < 0.001; I^2^ = 95.4%
Follow-up duration				
≥2 years	3	1.84	1.24–2.73	p < 0.001; I^2^ = 96.1%
<2 years	3	1.55	1.33–1.80	p = 0.349; I^2^ = 5.0%
Type of patients				
Dialysis	2	1.58	1.36–1.84	p = 0.620; I^2^ = 0.0%
Non-dialysis CKD	4	1.68	1.16–2.44	p < 0.001; I^2^ = 94.7%

Abbreviations: CKD, chronic kidney disease; HR, hazard ratio; CI, confidence interval; NOS, Newcastle-Ottawa Scale.

**Table 4:** Subgroup analysis of hypernatraemia and all-cause mortality.

Subgroup	No. of studies	Pooled HR	95%CI	Heterogeneity between studies
**1. Baseline hypernatraemia**				
Sample sizes				
≥10,000	3	1.04	0.84–1.30	p < 0.001; I^2^ = 88.1%;
<10,000	3	1.30	0.91–1.86	p = 0.117; I^2^ = 53.5%
Study design				
Prospective	2	1.46	0.85–2.52	p = 0.066; I^2^ = 70.3%;
Retrospective	4	1.04	0.86–1.86	p = 0.001; I^2^ = 82.2%
Follow-up duration				
≥2 years	3	1.15	0.95–1.39	p = 0.012; I^2^ = 77.3%
<2 years	3	1.15	0.70–1.89	p = 0.008; I^2^ = 80.9%
Type of patients				
Dialysis	2	0.86	0.74–1.01	p = 0.419; I^2^ = 0.0%
Non-dialysis CKD	4	1.23	0.99–1.52	p = 0.003; I^2^ = 78.8%
**2. Time-averaged hypernatraemia**				
Sample sizes				
≥10,000	3	1.42	1.16–1.75	p < 0.001; I^2^ = 87.8%;
<10,000	3	1.37	1.04–1.82	p = 0.707; I^2^ = 0.0%
Study design				
Prospective	2	1.5	1.06–2.12	p = 0.910; I^2^ = 0.0%;
Retrospective	4	1.39	1.15–1.68	p = 0.001; I^2^ = 82.0%
Follow-up duration				
≥2 years	3	1.42	1.08–1.85	p = 0.001; I^2^ = 86.6%
<2 years	3	1.45	1.26–1.66	p = 0.650; I^2^ = 0.0%
Type of patients				
Dialysis	2	1.44	1.24–1.66	p = 0.368; I^2^ = 0.0%
Non-dialysis CKD	4	1.43	1.13–1.81	p = 0.002; I^2^ = 80.3%

Abbreviations: CKD, chronic kidney disease; HR, hazard ratio; CI, confidence interval; NOS, Newcastle-Ottawa Scale.

## Discussion

This is the first meta-analysis that investigated the association of dysnatremia with all-cause mortality risk. From the seven included observational studies, we found that baseline hyponatremia and time-dependent hyponatremia or hypernatremia were independently associated with increased risk of all-cause mortality in CKD patients. The risk of all-cause mortality for CKD patients who have time-dependent hyponatremia or hypernatremia increased by 41% and 65%, respectively; while the risk of all-cause mortality for CKD patients who had hyponatremia at baseline increased by 34%. However, the association of baseline hypernatremia with all-cause mortality was not statistically significant. Taken together, our results suggest that the association of serum sodium level with all-cause mortality appeared to exhibit a U-shaped trend in CKD patients. Therefore, dysnatremia may have potential in mortality risk stratification among these patients.

A previous meta-analysis revealed that moderate hyponatremia was associated with an increased 1.60-fold risk of overall mortality^20^. However, this meta-analysis enrolled patients with myocardial infarction, heart failure, cirrhosis, pulmonary infection, or mixed diseases, and did not concentrate on CKD patients. Our meta-analysis was specially focused on CKD patients. We found that both hyponatremia and hypernatremia were strongly associated with higher risk of all-cause mortality when analyzed in a time-dependent manner. However, the combined risk estimate was not statistically significant when measured as a single baseline sodium level. Particularly, time-averaged hypernatremia was more strongly associated with all-cause mortality than single baseline hypernatremia. Water and sodium removal were almost exclusively determined by the dialysis procedure in ESRD patients. CKD patients undergoing dialysis are prone to develop dysnatremia. Therefore, the severity of the kidney disease may affect the mortality associated with dysnatraemia. In our subgroup analyses, patients undergoing dialysis exhibited a relatively higher all-cause mortality (HR: 1.27 *vs*. 1.41) associated with baseline hyponatremia, compared with patients with non-dialysis CKD. In contrast, the stage of CKD did not appear to affect the mortality associated with hypernatremia.

The prevalence of hyponatremia was significantly higher at all stages of CKD than the prevalence of hypernatremia^6^. A number of studies^15–17,21^ that examined the association of serum sodium with all-cause mortality risk have focused largely on hyponatremia. Irrespective of the severity of kidney disease, all studies found that hyponatremia was associated with increased risk of all-cause mortality. Each 4 mmol/L increase in baseline sodium level was associated with 19–28% lower risk of all-cause mortality in maintenance hemodialysis patients^22,23^. As for the noted U-shape association of sodium level with mortality in CKD patients, elevated serum sodium levels also conferred to higher risk of all-cause mortality. The above mentioned studies selected the highest sodium level as the reference control. Thus, the association between hyponatremia and mortality in these studies might have been underestimated.

The exact mechanism of dysnatraemia in all-cause mortality risk in CKD patients remains unclear. Kidneys are responsible for maintaining water homeostasis, and CKD could magnify the effect of dysnatremias on their clinical consequences^24,25^. Both hyponatremia and hypernatremia can have direct adverse effects on the function of various organs, including the brain, heart, or musculoskeletal system; and subsequently, increase mortality.

For patients with renal disease, hyponatremia at baseline and time-dependent hyponatremia or hypernatremia had higher risk of mortality. Dysnatremia may be a potential target for correction for clinicians. However, whether correct dysnatraemia could improve outcomes should be evaluated in future studies.

Several limitations of this meta-analysis should be acknowledged. First, the number of included studies was relatively limited. Thus, the results of these subgroup analyses may not be robust. Second, there was statistical heterogeneity in the quantitative pooling outcome. Potential explanations for heterogeneity included the severity of the disease, follow-up duration and study design. Third, the measurement of serum sodium levels at a single time point could have resulted in the misclassification of dysnatremia due to the fluctuation of sodium values between dialysis sessions. However, time-averaged sodium analysis further confirmed the association of dysnatremia with all-cause mortality. A mean value of several monthly measurements of sodium may have provided more accurate results of dysnatremia. Fourth, the adjusted confounding factors were different across these included studies, and the lack of adjustment for these confounding factors may have slightly overestimated the risk estimate. Fifth, the lack of information on the dialysis regimen of these included studies was a potential limitation. Finally, this meta-analysis could not distinguish the effect of dysnatremia on peritoneal dialysis and hemodialysis.

In conclusion, baseline hyponatremia and time-dependent hyponatremia/hypernatremia are independently associated with increased all-cause mortality risk in CKD patients. This meta-analysis suggests the U-shaped association of dysnatremia with all-cause mortality risk in CKD patients. However, whether correcting dysnatremia can reduce mortality risk in CKD patients needs to be investigated in the randomized controlled trials.

## Methods

### Literature Search

The present meta-analysis was conducted in accordance with the checklists of the Meta-analysis Of Observational Studies in Epidemiology statement^26^. Two authors (LG Sun and Y Hou) independently searched the Web of Science, PubMed and Embase databases for available observational studies from inception to March 2017. The following search terms were used: (chronic kidney disease OR end-stage renal disease OR hemodialysis OR peritoneal dialysis) AND (sodium OR hyponatraemia OR hypernatremia OR dysnatraemia) AND (mortality OR death). In addition, a manual search of the reference lists of all relevant articles was performed to identify any additional eligible publications.

### Study Selection

Studies that satisfied the following criteria were eligible: (1) prospective or retrospective observational studies; (2) study populations that comprised of dialysis or non-dialysis CKD patients; (3) the exposure was baseline and time-averaged hyponatremia or hypernatremia; (4) the outcome measure was all-cause mortality; (5) the reported multiple adjusted HR and 95% CI compared hyponatremia or hypernatremia with normonatremia. Exclusion criteria: (1) studies that used the highest serum sodium level as reference controls; (2) serum sodium levels were used as the continuous variable; (3) studies that had a follow-up duration of less than six months.

### Data extraction and quality assessment

Two authors independently abstracted the following information into standardized forms: surname of the first author, publication year, country of origin, study design, sample size, percentage of male patients, age at enrollment, baseline estimated glomerular filtration rate (eGFR), hyponatremia or hypernatremia definition and number of patients, number of deaths, the most fully adjusted risk estimate, follow-up duration, and adjustment for potential cofounders. Any differences in opinion in the data extraction were resolved by discussion. In evaluating for methodological quality, the NOS was used for these cohort studies^27^. The NOS was based on the following three aspects: selection, comparability and outcome. When using this scale, a total score of ≥7 stars was deemed of high quality.

### Data analysis

The pooled risk estimate was calculated through the category of hyponatremia or hypernatremia *vs*. normonatremia in these individual studies. Heterogeneity was explored using the Cochrane *Q* test and *I* ^2^ statistic. Statistical heterogeneity was set at an *I* ^2^ statistic of ≥50% and/or *Q* test of *P* < 0.10. In the presence of statistical heterogeneity, studies were pooled using a random effect model. Otherwise, a fixed-effect model was used. Subgroup analyses were performed according to the type of patients (dialysis *vs*. non-dialysis CKD), study design (prospective *vs*. retrospective), sample size (≥10,000 *vs*. <10,000) and follow-up duration (≥2 years *vs*. <2 years). A sensitivity analysis was conducted to investigate the impact of these individual studies on the overall results by removing one study at each turn. Potential publication bias was explored using Begg’s test and Egger’s test. All statistical analyses were performed using STATA software 12.0 (Stata Corp, College Station, Texas).
